# CysK2 couples copper sensing to redox adaptation in *Mycobacterium tuberculosis*

**DOI:** 10.1128/mbio.00816-26

**Published:** 2026-09-08

**Authors:** Wendy Le Mouëllic, Florence Levillain, Ting-Di Wu, Gérald Larrouy-Maumus, Frédéric Lagarrigue, Edith Vidal, Yoann Rombouts, Yannick Poquet, Olivier Neyrolles

**Affiliations:** 1 Univ Toulouse, CNRS, IPBS 54920, Toulouse, France; 2 Institut Curie, CNRS Unité d'Appui et de Recherche UAR2016, Inserm Unité de Service US43, Université Paris-Saclay, Multimodal Imaging Center 55216, Orsay, France; 3 Centre for Bacterial Resistance Biology, Department of Life Sciences, Faculty of Natural Sciences, Imperial College London 4615, London, United Kingdom; Washington University in St. Louis, St. Louis, Missouri, USA

**Keywords:** *Mycobacterium tuberculosis*, copper sensing, cysteine biosynthesis, redox metabolism, persistence

## Abstract

Copper is recognized as a host-derived cue encountered by *Mycobacterium tuberculosis* and other microbes during infections, yet the magnitude, intracellular distribution, and physiological consequences of this exposure remain incompletely understood. Here, we combined high-resolution imaging, transcriptomic profiling, intracellular reporter assays, and mouse infection models to define how *M. tuberculosis* responds to physiologically relevant copper levels during infection. NanoSIMS analysis showed that copper reaches intracellular bacilli and accumulates in discrete phosphorus-rich foci in bacteria. Exposure to physiological copper concentrations *in vitro* triggered a highly specific transcriptional response dominated by the copper-inducible CsoR and RicR regulons. The RicR-regulated *cysK2* gene, encoding the (*S*-sulfo)cysteine synthase CysK2, was one of the most strongly induced loci. In infected macrophages, *cysK2* expression was modulated by extracellular copper availability, host copper transport pathways, and hypoxia. *In vivo*, the H37Rv *cysK2*-deficient mutant showed reduced long-term persistence in mice and a shift toward a more oxidized redox potential. Together, these findings identify CysK2 as a copper-responsive effector linking host-derived copper sensing to redox homeostasis and contributing to long-term persistence during infection.

**IMPORTANCE:**

Successful infection by *Mycobacterium tuberculosis* depends on its ability to detect and adapt to host-imposed changes in the phagosomal environment. Our work shows that copper contributes to this adaptation not simply by imposing toxicity, but by triggering a response that helps sustain bacterial fitness within host cells. We identify CysK2 as a key component of this response, linking copper sensing to sulfur metabolism, redox balance, and persistence during infection. These findings shift the view of copper from a purely antimicrobial factor to a host-derived environmental signal that remodels *M. tuberculosis* physiology, and they uncover a new mechanism by which the pathogen maintains intracellular survival.

## INTRODUCTION

Tuberculosis (TB), caused by *Mycobacterium tuberculosis*, remains one of the world’s deadliest infectious diseases. In 2024, an estimated 10.7 million new cases and 1.23 million deaths were reported worldwide (1). Despite decades of intensive research and the availability of antimicrobial therapy, *M. tuberculosis* continues to pose a profound global health burden. Its success as a pathogen depends in part on its ability to withstand host-imposed stresses and to remodel its metabolism during infection. Defining these adaptive mechanisms may reveal vulnerabilities that can be exploited to improve TB treatment.

Successful persistence of *M. tuberculosis* within the host requires continuous adaptation to the restrictive intracellular environment, particularly within macrophages. In the phagosome, *M. tuberculosis* encounters multiple stresses, including toxic antimicrobial factors and limitation of essential nutrients (2, 3). Metal ions represent a particularly acute challenge because they are indispensable for bacterial physiology yet become toxic when present in excess. Accordingly, *M. tuberculosis* must tightly control intracellular metal homeostasis, while macrophages exploit metal imbalance as an antimicrobial strategy by withholding essential metals or promoting their toxic accumulation (4–6).

Among metal-dependent host defenses, copper has emerged as a contributor to macrophage antimicrobial activity, notably against *Escherichia coli* and *Salmonella enterica* serovar Typhimurium (7, 8). In macrophages, copper uptake is mediated by CTR1, whereas its intracellular distribution is controlled by copper chaperones such as ATOX1 and by the copper-transporting ATPase ATP7A (9–12). Under activating conditions, including lipopolysaccharide (LPS)/interferon (IFN)-γ stimulation, *Salmonella* infection, and hypoxia, *Ctr1* and *Atp7a* expression increases, intracellular copper accumulates and is redistributed, while ATP7A relocalizes from the Golgi to vesicular compartments that can associate with pathogen-containing phagosomes (7, 8, 13, 14).

In TB, several observations are consistent with a role for copper during infection. Copper supplementation in drinking water attenuates pulmonary pathology in *M. tuberculosis*-infected mice, although it does not reduce bacterial burden, and copper accumulates within granulomas in the lungs of infected guinea pigs (15). At the cellular level, however, copper dynamics in *M. tuberculosis*-infected macrophages remains poorly defined, as only a single study has reported a transient increase in copper within *M. tuberculosis*-containing phagosomes early after infection (16).

At the bacterial level, the response of *M. tuberculosis* to copper stress has been extensively characterized *in vitro* (17–21). Copper induces a transcriptional program controlled primarily by the copper-responsive repressors CsoR and RicR (22, 23). Notably, the CsoR regulon includes an operon encoding CsoR itself, together with CtpV, a putative copper-exporting P-type ATPase, and PacL3, a cognate metal-binding chaperone potentially implicated in resistance to copper intoxication (22–26). However, our recent study did not support a role for the PacL3-CtpV module in copper resistance under the conditions tested (27). The RicR regulon encodes several proteins that contribute to *M. tuberculosis* resistance to high copper concentrations *in vitro*, including the metallothionein MymT and the multicopper oxidase MmcO (22, 28, 29). However, whereas constitutive repression of the entire RicR regulon attenuates *M. tuberculosis in vivo* (30), deletion of *mymT*, *mmcO*, or both does not impair bacterial fitness in animal models (28, 30). This discrepancy highlights that the role of copper, and the consequences of copper exposure for *M. tuberculosis* during infection, remain less well understood *in vivo* than under defined *in vitro* conditions.

Among the most strongly copper-induced loci in the RicR regulon is the *lpqS-cysK2-rv0849-rv0850* operon, whose function remains poorly understood (22). Of the four predicted gene products encoded by this locus, only an *lpqS* mutant has been examined, and it showed no detectable phenotype under copper stress (30). The downstream-encoded enzyme CysK2 was initially characterized as an *O*-phosphoserine-dependent cysteine/*S*-sulfocysteine synthase (31), but its contribution to copper adaptation had not been tested.

In this study, we investigated how copper encountered during macrophage infection shapes the adaptation of *M. tuberculosis*. We show that copper reaches intracellular bacilli and induces expression of *cysK2*. Using redox reporter strains and mouse models, we demonstrate that CysK2 preserves redox homeostasis and promotes bacterial persistence during late-stage infection. Collectively, our findings refine our understanding of copper exposure during *M. tuberculosis* infection and identify CysK2 as a copper-responsive node linking host-derived copper sensing to redox homeostasis and persistence *in vivo*.

## RESULTS

### Copper accumulates in discrete phosphorus-rich foci within intracellular *M. tuberculosis*

Copper accumulation in pathogen-containing vacuoles is widely viewed as part of the macrophage antimicrobial response. However, direct visualization of copper within *M. tuberculosis*-containing compartments is limited. Previous X-ray fluorescence (XRF)-based analysis detected copper in mycobacterial phagosomes, but its spatial resolution did not allow precise subcellular localization within infected cells or bacilli (16).

**Fig 1:**
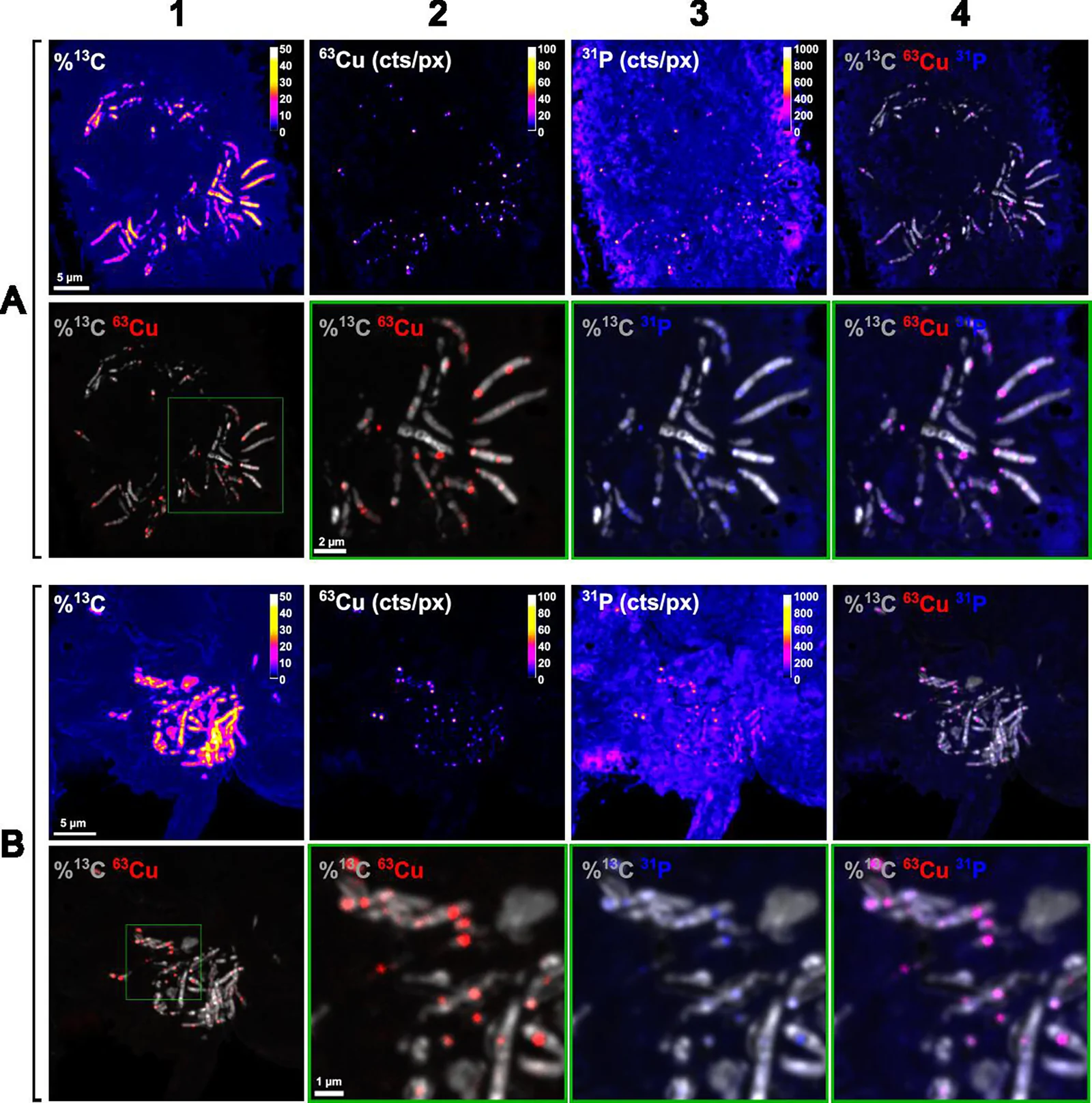
Copper accumulates in discrete phosphorus-rich foci within intracellular *M. tuberculosis*. Murine bone marrow-derived macrophages were infected with *M. tuberculosis* previously cultured in 7H9 supplemented with ^13^C-glycerol. Following a 4-h internalization period, extracellular bacteria were removed by washing, and culture medium + 200 µM CuCl_2_ was added. Three days post-infection, cells were fixed with 4% paraformaldehyde and analyzed by nanoSIMS to visualize the distribution of ^13^C, ^63^Cu, and ^31^P in host cells and intracellular bacilli. (**A** and **B**) Representative images from two infected macrophages. % ^13^C represents ^13^C atom fraction (%) calculated as ^13^C/(^13^C + ^12^C) × 100. The ^63^Cu and ^31^P signals represent the number of counts per pixel (cts/px).

To determine whether copper reaches intracellular *M. tuberculosis* and to define its subcellular distribution in infected macrophages, we performed high-resolution nanoSIMS imaging. Murine bone marrow-derived macrophages were infected with ^13^C-labeled *M. tuberculosis*, allowing simultaneous detection of copper and ^13^C-enriched bacterial regions. Three days post-infection, we did not detect copper enrichment in the host cell cytosol (Fig. 1, columns 1 and 2). In contrast, copper accumulation was apparent within ^13^C-enriched bacterial regions (Fig. 1, columns 1 and 2). Copper distribution within bacilli was heterogeneous, with most intracellular bacteria containing one or several highly enriched foci located at both polar and non-polar positions. These foci colocalized with phosphorus-rich areas (Fig. 1, columns 3 and 4), which may correspond to polyphosphate granules, long described in *M. tuberculosis* (32). Together, these data indicate that, under these conditions, extracellularly supplied copper can reach intracellular *M. tuberculosis* and accumulate in discrete phosphorus-rich intrabacterial foci.

### Physiological copper levels trigger strong *cysK2* induction in *M. tuberculosis*

Having established that copper can reach intracellular *M. tuberculosis*, we next asked how these physiological copper levels affected bacterial gene expression. Previous transcriptomic studies examined the response of *M. tuberculosis* to moderate-to-high copper concentrations *in vitro*, but the effects of lower concentrations, such as those reported in phagosomes at 24 h post-infection (16), were unknown. We therefore performed RNA-seq analysis on bacilli exposed to either 20 µM copper sulfate, chosen to approximate physiological exposure (33, 34), or 100 µM (high) copper.

**Fig 2:**
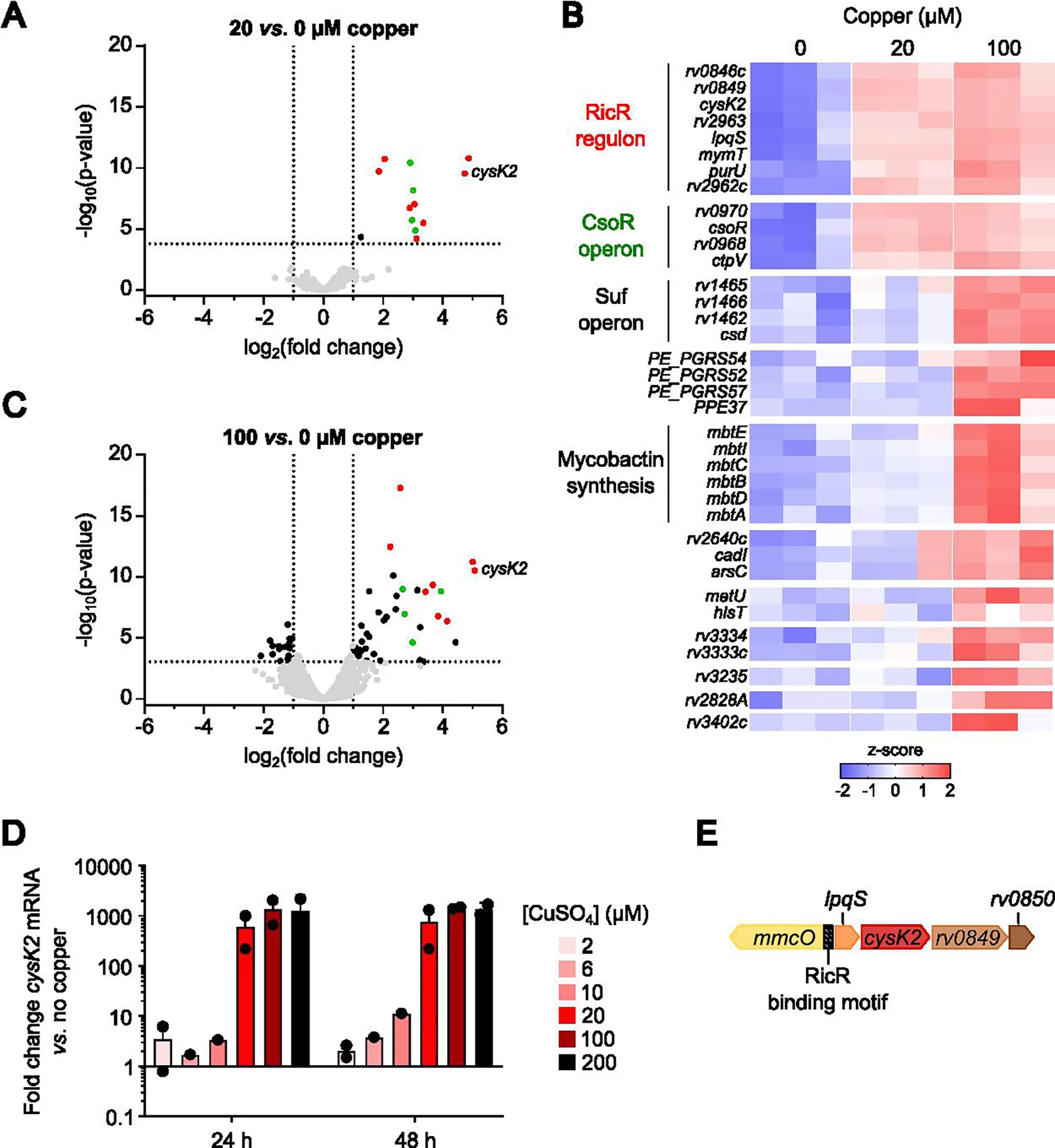
Physiological copper concentrations trigger a focused transcriptional response and strongly induce *cysK2*. Total RNA was isolated from *M. tuberculosis* cultures grown to an OD_600_ of 0.8 in the presence of 0, 20, or 100 µM copper and subjected to RNA-seq analysis. (**A**) Volcano plot showing the distribution of genes according to log_2_(fold change) and −log_10_(*P* value) in 20 µM vs 0 µM copper condition. (**B**) Heatmaps showing expression changes across selected functional categories. Heatmaps were generated using row *z*-scores of normalized logCPM values. (**C**) Volcano plot showing the distribution of genes according to log_2_(fold change) and −log_10_(*P* value) in 100 µM vs 0 µM copper condition. (**D**) *M. tuberculosis* was incubated in M9 minimal medium supplemented with 0, 2, 6, 10, 20, 100, or 200 µM CuSO_4_. After 24 or 48 h, total RNA was extracted, and *cysK2* expression was quantified by RT-qPCR. Data represent mean ± SD fold change relative to the reference gene *rpoB* and the 0 µM CuSO_4_ condition from two independent experiments. (**E**) Genetic organization of the *cysK2* locus in *M. tuberculosis*.

Exposure to 20 µM copper elicited a highly specific transcriptional response (Fig. 2A), with nearly all genes induced at 20 µM copper belonging to the CsoR and RicR regulons (Fig. 2B). By contrast, 100 µM copper induced a broad stress response involving more than 50 genes, mostly involved in Fe-S cluster biogenesis, mycobactin biosynthesis, and cell envelope-associated functions (Fig. 2B and C).

Among the genes most strongly induced at 20 µM copper were *mymT*, *mmcO*, *ctpV*, and *cysK2* (*rv0848*). Because the role of CysK2 in copper adaptation had not been previously investigated, we examined its regulation in more detail. RT-qPCR analysis showed that *cysK2* expression increased approximately 4-fold after exposure to as little as 6 µM copper for 48 h and reached substantial induction, exceeding 500-fold, at 20 µM copper (Fig. 2D). Consistently, a dual reporter strain carrying a constitutive GFP and *lpqSp*-mCherry confirmed *lpqS*/*cysK2* promoter activation at copper concentrations as low as 6 µM (Fig. S1A).

In agreement with a previous report (22), and with the organization of *cysK2* within the RicR-regulated operon (Fig. 2E), other operon-associated genes, including *lpqS*, *rv0849*, and the upstream gene *mmcO*, were also induced by copper (Fig. S1B). We then asked whether this response extended more broadly to sulfur assimilation and cysteine biosynthesis pathways. However, genes encoding the sulfate transporter SubI (35, 36), the alternative cysteine synthases CysK1 and CysM (37–39), and the reverse transsulfuration enzyme MetB (40) did not show induction comparable to *cysK2* (Fig. S1B). Thus, physiological copper exposure does not broadly induce sulfur metabolic pathways in *M. tuberculosis*, but instead elicits a focused transcriptional response in which CysK2 emerges as a uniquely and strongly induced component of the sulfur metabolic network.

### *cysK2* is genomically associated with copper-homeostasis loci in Actinomycetes

**Fig 3:**
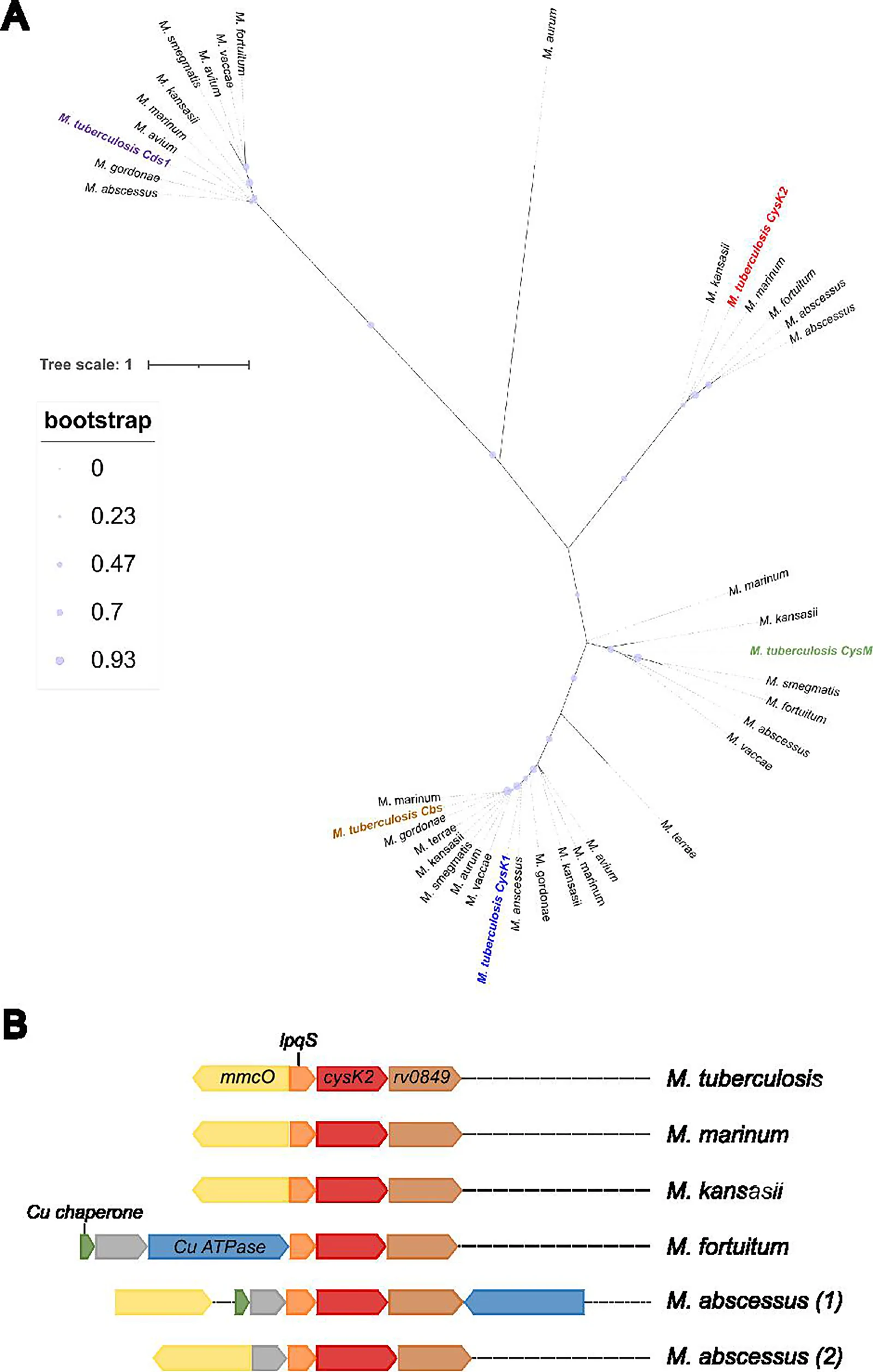
Phylogenetic distribution and genomic context of CysK2-related proteins in Actinomycetes. (**A**) Phylogenetic tree of selected proteins related to the *M. tuberculosis* CysM, CysK1, Cbs, CysK2, and Cds1. The *M. tuberculosis* reference proteins are highlighted in color: CysM (green), CysK1 (blue), Cbs (maroon), CysK2 (red), and Cds1 (purple). Representative homologs from mycobacteria are shown. Circle size indicates bootstrap support, as shown in the inset. The scale bar represents 1 substitution per 100 amino acid positions. Protein accession numbers (UniProt) used to construct the tree are listed in Table S3. (**B**) Representative genomic neighborhoods of *cysK2*-containing loci in selected mycobacteria. Gene arrows indicate predicted coding sequences and transcriptional orientation. Color coding highlights conserved functional categories associated with the locus: *cysK2* (red), *lpqS* (orange), *rv0849* (brown), *mmcO* (yellow), genes encoding copper ATPases (blue), and copper chaperones (green).

To further explore the potential function of CysK2, we examined its phylogenetic distribution across bacterial phyla using BLAST searches in the UniProt database. Homologs sharing more than 50% amino acid identity with *M. tuberculosis* CysK2 were detected exclusively within Actinomycetes. Within this class, homologs were most frequently found in Kitasatosporales, Mycobacteriales, Pseudonocardiales, and Streptosporangiales, were less common in Micromonosporales, and were rare or absent in other actinomycete lineages (Table S1). In a representative panel of *Mycobacterium* species spanning rapid- and slow-growing taxa as well as pathogenic, opportunistic, and non-pathogenic species, CysK2 formed a distinct clade separate from the *M. tuberculosis* sulfur-metabolism enzymes CysK1, CysM, Cbs, and Cds1 (Fig. 3A). Within this set, CysK2 was present in species including *M. marinum*, *M. kansasii*, *M. fortuitum*, and *M. abscessus*, but absent from others, including *M. avium*, *M. smegmatis*, *M. gordonae*, *M. aurum*, *M. terrae*, and *M. vaccae* (Fig. 3A). Thus, its presence did not segregate clearly with growth rate or pathogenicity.

We next examined the genomic context of *cysK2* in the selected CysK2-containing *Mycobacterium* species using the Integrated Microbial Genomes and Microbiomes database. In all species examined, *cysK2* was associated with a conserved copper-related locus (Fig. 3B). Together, these analyses identify *cysK2* as a lineage-restricted actinomycete gene recurrently associated with copper-related genomic neighborhoods. Combined with its strong induction by physiological copper levels in *M. tuberculosis*, this organization supports a role for CysK2 in copper-responsive adaptation.

### Host-derived copper triggers *cysK2* expression in intracellular *M. tuberculosis*

**Fig 4:**
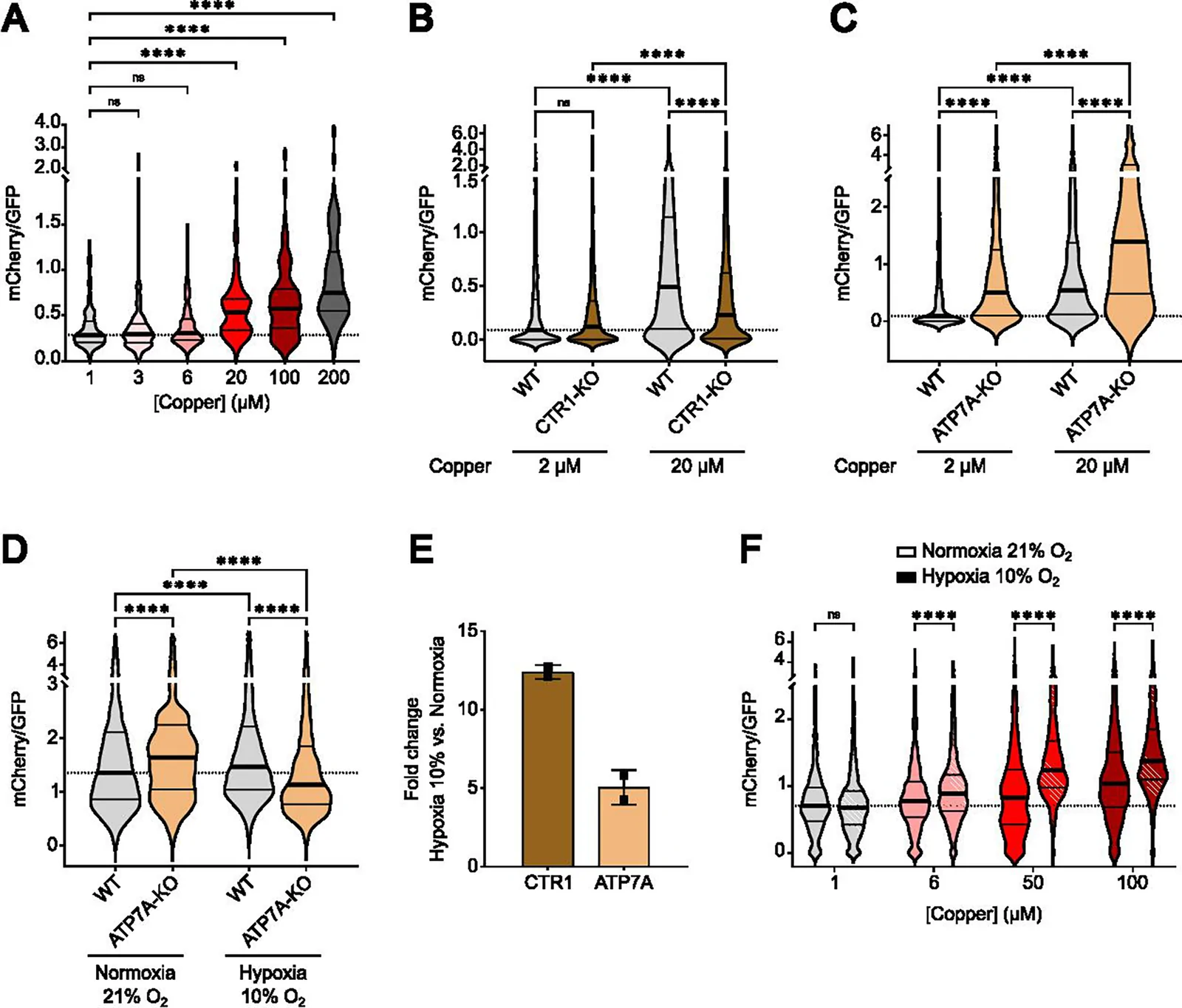
Host-derived copper induces *cysK2* expression in intracellular *M. tuberculosis*. (**A**) Murine bone marrow-derived macrophages (BMDMs) were infected with the *M. tuberculosis* P1-GFP *lpqSp-*mCherry reporter strain in the presence of increasing extracellular copper concentrations (CuCl_2_). At 4 days post-infection, cells were fixed, and *lpqSp* activity in intracellular bacilli was quantified by confocal microscopy as the mCherry/GFP fluorescence ratio. Data represent violin plots showing the median and interquartile range of *n* = 38–264 bacteria per condition. Measurements from bacteria imaged in three replicate wells were pooled for visualization. Statistical test: Kruskal–Wallis test + Dunn’s *post hoc* test. (**B and C**) Wild-type, CTR1-knockout, or ATP7A-knockout HoxB8-derived macrophages were infected with the reporter strain under basal copper conditions (2 µM, contributed by 10% FBS) or after supplementation with 20 µM CuSO_4_. At 6 days post-infection, *lpqSp* activity in intracellular bacilli was quantified as the mCherry/GFP fluorescence ratio. Data represent violin plots showing the median and interquartile range of *n* = 739–3,735 bacteria per condition. Measurements from bacteria imaged in three replicate wells were pooled for visualization. Statistical test: Kruskal–Wallis test + Dunn’s *post hoc* test. (**D**) Wild-type and ATP7A-knockout HoxB8-derived macrophages were infected with the reporter strain and cultured with 20 µM CuSO_4_ under normoxic (21% O_2_) or hypoxic (10% O_2_) conditions. At 6 days post-infection, *lpqSp* activity in intracellular bacilli was quantified as the mCherry/GFP fluorescence ratio. Data represent violin plots showing the median and interquartile range of *n* = 3,365–8,789 bacteria per condition. Measurements from bacteria imaged in three replicate wells were pooled for visualization. Statistical test: Kruskal–Wallis test + Dunn’s *post hoc* test. (**E**) BMDMs were activated with IFN-γ and LPS and cultured under normoxic (21% O_2_) or hypoxic (10% O_2_) conditions for 3 days. Total RNA was extracted, and *Ctr1* and *Atp7a* expression was quantified by RT-qPCR. Data represent mean ± SD from two independent RNA extractions. (**F**) Murine BMDMs were infected with the reporter strain in the presence of increasing extracellular copper concentrations (CuSO_4_) under normoxic (21% O_2_) or hypoxic (10% O_2_) conditions. At 4 days post-infection, *lpqSp* activity in intracellular bacilli was quantified by confocal microscopy as the mCherry/GFP fluorescence ratio. Data represent violin plots showing the median and interquartile range of *n* = 89–1,892 bacteria per condition. Measurements from bacteria imaged in three replicate wells were pooled for visualization. Statistical test: Kruskal–Wallis test + Dunn’s *post hoc* test. ns, not significant; ****, *P* < 0.0001.

We next tested if intracellular copper levels can modulate *cysK2* expression during macrophage infection. Murine macrophages were infected with the *lpqSp*-mCherry reporter strain and increasing concentrations of copper were added to culture media after bacterial internalization. Four days post-infection, *lpqSp* activity was quantified as the mCherry/GFP fluorescence ratio in intracellular bacilli. No significant increase in reporter activity was detected below 20 µM extracellular copper, whereas higher concentrations triggered a progressive increase in *lpqSp* activity that did not saturate even at 200 µM, corresponding to the higher concentration suitable with good macrophage viability (Fig. 4A). Thus, in contrast to the strong and saturable induction observed in broth culture at concentrations as low as 6 µM copper (Fig. S1A), these data indicate that only a fraction of extracellular copper reaches intracellular bacilli during macrophage infection.

To determine whether this response depends on host copper trafficking, we infected wild-type, CTR1-, or ATP7A-KO HoxB8-derived macrophages (Fig. S2) with the reporter strain and measured *lpqSp* activity under basal (2 µM) or physiological (20 µM) copper conditions. Six days post-infection, copper supplementation increased reporter activity in all three cell lines (Fig. 4B and C), consistent with the results obtained in primary macrophages. However, *lpqSp* activity was reduced in intracellular bacilli residing in CTR1-deficient macrophages compared with wild-type cells (Fig. 4B), in line with the role of CTR1 in extracellular copper import. By contrast, bacilli infecting ATP7A-deficient macrophages displayed higher reporter activity than those in wild-type cells under the same conditions (Fig. 4C), indicating that ATP7A influences the copper-responsive signal perceived by intracellular bacteria in a reverse manner. Together, these results show that host-derived copper can induce *cysK2* expression in intracellular *M. tuberculosis* and that this response is modulated by macrophage copper transport pathways.

### Hypoxia enhances host-dependent copper signaling to intracellular *M. tuberculosis*

Because ATP7A relocalizes in response to conditions associated with macrophage activation or hypoxia (14), a feature of the deep lung and of TB lesions, we next examined its contribution under reduced oxygen tension. We infected wild-type and ATP7A-deficient macrophages with the *lpqSp* reporter strain and cultured at 10% O_2_, a level comparable to that reported in lung tissue (41). Under these conditions, reporter activity was lower in bacilli residing in ATP7A-deficient macrophages than in wild-type cells (Fig. 4D), indicating that ATP7A was required for maximal copper-dependent *cysK2* induction under hypoxia.

Additionally, we showed that 10% O_2_ increased *Ctr1* and *Atp7a* expression in LPS/IFN-γ-activated primary macrophages relative to normoxia (Fig. 4E). Consistent with the effect of hypoxia on copper transporter expression and ATP7A relocalization (14), infected macrophages cultured at 10% O_2_ showed increased *lpqSp* activity compared with normoxic controls at the same extracellular copper concentration (Fig. 4F). By contrast, planktonic bacilli exposed to 10% O_2_ did not show increased reporter activity across copper concentrations (Fig. S3), indicating that the effect of hypoxia is mediated by the host rather than by a direct bacterial response to this oxygen level. These results indicate that moderate tissue-like hypoxia enhances copper-dependent *cysK2* induction in intracellular *M. tuberculosis* through a host-dependent mechanism to which ATP7A contributes.

### CysK2 promotes redox homeostasis and persistence of *M. tuberculosis in vivo*

**Fig 5:**
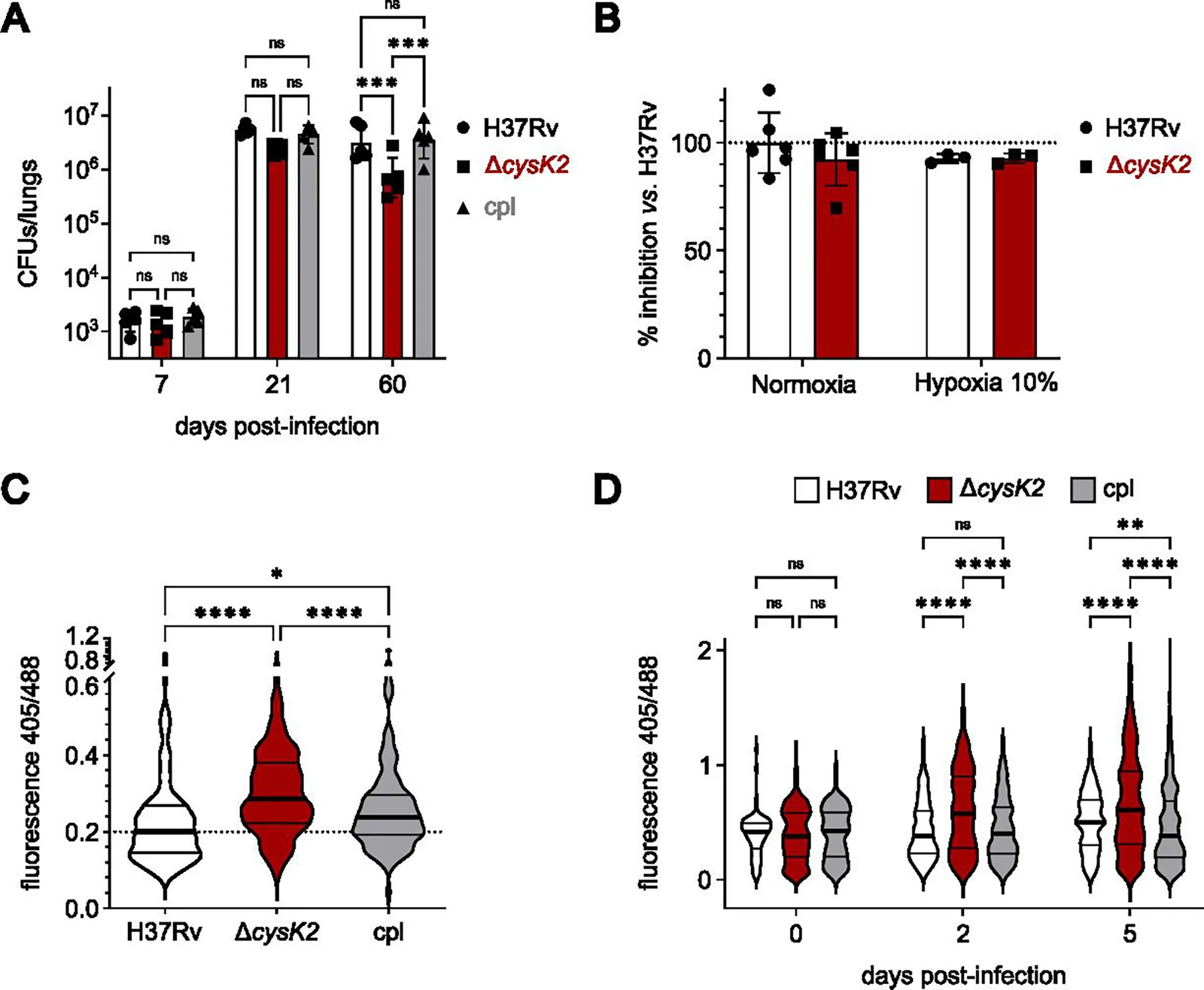
CysK2 contributes to *M. tuberculosis* persistence and redox homeostasis during infection. (**A**) C3HeB/FeJ mice were infected by aerosol with H37Rv, Δ*cysK2*, or the complemented strain (~100 CFU per mouse). Lungs were collected at 7, 21, and 60 days post-infection, and bacterial burden was quantified by CFU enumeration. Data represent mean ± SD for *n* = 5 mice per strain. Statistical analysis: two-way ANOVA with Tukey’s *post hoc* test on log-transformed data. Data show one representative experiment out of two independent experiments. (**B**) Copper sensitivity of H37Rv and Δ*cysK2* strains was assessed by disk diffusion assay using 5 µL of 1 M CuSO_4_. Plates were incubated under normoxic (21% O_2_) or hypoxic (10% O_2_) conditions, and inhibition zones were measured and normalized to the H37Rv wild-type strain under normoxia. Data represent mean ± SD of three or six biological replicates from two independent experiments. (**C**) C3HeB/FeJ mice were infected with H37Rv, Δ*cysK2*, and complemented strains expressing the Mrx1-roGFP redox reporter. At 60 days post-infection, lungs were collected and analyzed by confocal microscopy. The 405/488 fluorescence ratio was quantified in individual bacteria as a measure of the mycothiol redox potential. Violin plots show median and interquartile range for *n* = 115 (H37Rv), *n* = 341 (Δ*cysK2* mutant), and *n* = 124 (complemented strain) bacteria, pooled from two mouse samples. Statistical analysis: Kruskal–Wallis test, followed by Dunn’s multiple-comparison test. (**D**) Murine bone marrow-derived macrophages were infected with H37Rv, Δ*cysK2,* and complemented strains expressing the Mrx1-roGFP reporter. At the indicated times post-infection, cells were fixed and the 405/488 fluorescence ratio of intracellular bacteria was quantified by confocal microscopy. Violin plots show median and interquartile range for *n* = 347–11,191 individual bacteria, pooled from three independent wells. Statistical analysis: two-way ANOVA with Tukey’s *post hoc* test. ns, not significant; **, *P* < 0.01; ***, *P* < 0.001; ****, *P* < 0.0001.

To determine whether CysK2 contributes to *M. tuberculosis* virulence, we first assessed bacterial fitness during infection of susceptible C3HeB/FeJ mice, which develop necrotic granuloma-like structures that are not typically observed in C57BL/6 mice (42). We infected animals by aerosol with a low-to-medium bacterial dose (~100 CFU), and monitored pulmonary bacterial burdens over time (Fig. 5A). The Δ*cysK2* mutant (Fig. S4) showed moderate but significant attenuation during late-stage infection compared with wild-type and complemented strains, indicating that CysK2 contributes to bacterial persistence *in vivo*. To determine whether this phenotype was host-model specific, we performed parallel infections in resistant C57BL/6 mice, which confirmed a reproducible role for CysK2 in long-term infection (Fig. S5).

Because *cysK2* is strongly induced by copper during macrophage infection, particularly under tissue-like hypoxia, we next asked whether CysK2 contributes to copper resistance. We therefore compared the copper sensitivity of wild-type and Δ*cysK2* strains *in vitro* using disk diffusion assays (Fig. 5B), growth measurements in copper-supplemented rich medium (Fig. S6A), and CFU enumeration after copper exposure (Fig. S6B). Given that copper toxicity can be enhanced under low-oxygen conditions (43–46), we also tested sensitivity at 10% O_2_ (Fig. 5B). Under all conditions examined, the H37Rv Δ*cysK2* mutant behaved similarly to the wild type, suggesting that the *in vivo* attenuation of the Δ*cysK2* strain was not explained by increased copper sensitivity.

As CysK2 was identified as a (*S*-sulfo)cysteine synthase (31), we next investigated whether CysK2 contributes to redox homeostasis during infection. To assess the redox status *in vivo*, we generated wild-type, Δ*cysK2*, and complemented strains expressing Mrx1-roGFP, that reports modifications in the reduced mycothiol/oxidized mycothiol disulfide couple through changes in the 405/488 nm excitation ratio (47). We infected C3HeB/FeJ mice with these strains and analyzed lung sections by confocal microscopy 60 days post-infection (Fig. 5C). The 405/488 fluorescence ratio was significantly higher in Δc*ysK2* bacilli than in wild-type and complemented strains, indicating a shift toward a more oxidized mycothiol redox potential during chronic infection. Stratification of bacilli according to their localization in macrophage-rich cellular regions or acellular/necrotic lesions did not reveal a significant location-dependent difference in the reporter phenotype (Fig. S7). A similar redox imbalance was observed *ex vivo* in infected macrophages, where Δ*cysK2* bacteria exhibited a more oxidized state than wild-type and complemented strains as early as 2 days post-infection (Fig. 5D). Together, these data show that CysK2 promotes *M. tuberculosis* persistence during late-stage infection and supports redox homeostasis *in vivo* and in infected macrophages.

## DISCUSSION

Here, we show that host-derived copper reaches intracellular *M. tuberculosis* in macrophages and accumulates in discrete phosphorus-rich foci. Analysis of the transcriptional response of *M. tuberculosis* to moderate, physiologically relevant copper concentrations *in vitro* reveals a focused induction of CsoR and RicR regulons, including the *cysK2* gene. In macrophages, *cysK2* expression in intracellular bacilli is modulated by host copper transport and hypoxia. Finally, in H37Rv, loss of CysK2 does not confer overt copper hypersensitivity but shifts the redox balance toward a more oxidized state and reduces long-term persistence in mice.

We first sought to follow the path of copper from the extracellular environment to intracellular bacilli. Using high-resolution nanoSIMS, we found that copper reaches *M. tuberculosis* within infected macrophages and accumulates in discrete phosphorus-rich intrabacterial foci, consistent with localization within polyphosphate granules. To our knowledge, this is the first evidence that copper can accumulate within such structures in intracellular *M. tuberculosis*. Copper binding by polyphosphates has previously been described in other systems, including bacteria and the unicellular alga *Chlamydomonas* (48, 49). By contrast, previous scanning transmission electron microscopy (STEM)-based elemental analysis of polyphosphate granules in planktonic mycobacteria identified potassium, calcium, and magnesium but not copper (50). This discrepancy may reflect the different biological contexts examined, since our analysis was performed on intracellular bacilli, but it may also arise from technical differences in fixation and sample processing that affect retention of labile metal ions. Whether polyphosphates contribute directly to copper homeostasis in *M. tuberculosis* remains to be determined.

Our data also refine the current view of copper accumulation in the mycobacterial phagosome. Apart from these intrabacterial phosphorus-rich foci, we did not detect prominent copper enrichment in the surrounding compartment. This observation is in line with the earlier XRF study by Wagner et al. showing that phagosomal copper levels are modest at later time points after infection, reaching approximately 25 µM at 24 h (16). Importantly, the much higher copper concentration often cited in the literature corresponds to an early and highly variable transient peak measured 1 h after infection (426 ± 393 µM) (16). The transcriptional response of intracellular *M. tuberculosis* to copper is consistent with this model. Using an *lpqS/cysK2* promoter reporter, we show that intracellular *M. tuberculosis* responds to host-derived copper at levels sufficient to induce *cysK2*. In contrast to the rapid, saturable induction observed in broth, reporter activity in macrophages increased further with extracellular copper supplementation or hypoxia. These observations support the idea that only a fraction of extracellular copper is ultimately delivered to intracellular *M. tuberculosis*.

Our experiments using macrophages deficient in copper transporters begin to clarify how host copper trafficking influences the signal perceived by intracellular bacilli. Reduced *cysK2* induction observed in CTR1-deficient macrophages is consistent with the established role of CTR1 in copper import at the plasma membrane and supports a contribution of host-cell copper uptake to the signal perceived by intracellular *M. tuberculosis*.

The role of ATP7A appears more context-dependent. Under basal conditions, ATP7A deficiency increased reporter activity, suggesting that ATP7A may limit copper availability to bacilli in this context, possibly by redistributing copper to compartments distinct from the phagosome. Under moderate hypoxia, however, ATP7A was required for maximal *cysK2* induction. In the light of previous studies that showed ATP7A relocalization after exposure to severe hypoxia (14), we propose that tissue-like hypoxia similarly triggers ATP7A expression and redistribution, to modulate copper delivery to *M. tuberculosis*-containing phagosomes. Together, these observations indicate that copper trafficking to the mycobacterial compartment is dynamic and depends on the physiological state of the host cell rather than reflecting a fixed, uniformly high phagosomal copper concentration.

This context dependence may also be important when comparing copper biology across infection models. Tissue copper concentrations increase during infection, especially in the lung (51), but the magnitude and distribution of this increase may differ across species and lesion types. For example, copper accumulation has been reported in granulomas of guinea pigs during TB infection, whereas evidence for comparable lesion-level accumulation in mice is less clear (15, 52). Such differences could help explain why most copper-responsive genes that contribute to copper resistance *in vitro* or appear relevant in one animal model do not always show the same contribution in another.

Despite strong induction of *cysK2* by copper, the H37Rv Δ*cysK2* mutant did not display overt copper hypersensitivity under the conditions tested. A companion study by Ji et al. (53) provides a likely explanation: H37Rv carries a hypomorphic CysK2 G93 variant with substantially lower cysteine-synthase activity *in vitro* than the ancestral S93 enzyme found in Erdman and CDC1551. Accordingly, deletion of *cysK2* produces only a subtle copper-resistance phenotype in H37Rv, whereas the S93 variant confers robust copper resistance.

Nonetheless, our results show that loss of CysK2 is accompanied by a more oxidized intracellular state both *in vivo* and in infected macrophages. Thus, even though the *in vitro* activity of CysK2 is reduced in H37Rv compared to the Erdman and CDC1551 strains (53), CysK2-dependent cysteine production, strongly triggered by copper, seems sufficient to contribute significantly to H37Rv’s redox homeostasis during infection. It remains to be determined whether cysteine acts directly to control redox balance or as part of the biosynthesis pathways of essential redox buffers such as mycothiol and ergothioneine.

Finally, in two mouse models, loss of *cysK2* reduced long-term bacterial persistence. A recent study likewise linked CysK2 to *M. tuberculosis* survival in macrophages and in mice, although the magnitude of the *in vivo* phenotype was substantially greater than that observed here (54). The redox-centered role of CysK2 proposed here is consistent with the importance of sulfate uptake and sulfur assimilation for *M. tuberculosis* virulence (35, 55), and with the possibility that its multiple cysteine-biosynthetic pathways make distinct contributions under specific host conditions. CysK2 may therefore form part of a copper-responsive adaptation that helps preserve thiol-dependent redox balance during chronic infection.

## MATERIALS AND METHODS

### Bacterial strains and culture conditions

*Mycobacterium tuberculosis* strains were cultured at 37°C in Middlebrook 7H9 broth (Difco) supplemented with 10% albumin-dextrose-catalase (ADC; Difco) and 0.05% Tyloxapol (Sigma), or on Middlebrook 7H11 agar (Difco) supplemented with 10% oleic acid-albumin-dextrose-catalase (OADC; Difco). When appropriate, the culture medium was supplemented with hygromycin (50 µg/mL), streptomycin (25 µg/mL), kanamycin (20 µg/mL), or zeocin (25 µg/mL).

### Construction of *M. tuberculosis* mutant and complemented strain

The Δ*cysK2* mutant was generated in *M. tuberculosis* H37Rv by allelic exchange using recombineering with the primers listed in Table S2 as previously described (56).

The complementation plasmid pGMC-S-*lpqSp-lpqS-cysK2* was constructed by In-Fusion cloning (Takara) using pDE43-MCS as the destination vector and the primers listed in Table S2. The resulting plasmid was electroporated into the *M. tuberculosis* H37Rv Δ*cysK2*::dif4 strain carrying pJV53H.

Deletion of *cysK2* and complementation were verified by PCR on genomic DNA and by RT-qPCR on reverse-transcribed total RNA using specific primers (Table S2). For deletion, primers flanking the upstream and downstream regions of the locus were used; for complementation, primers internal to *cysK2* were used (Fig. S4).

### Construction of *M. tuberculosis* reporter strains

To generate the *cysK2* promoter reporter plasmid, an approximately 700 bp DNA fragment upstream of *lpqS* was PCR-amplified using the primers listed in Table S2 and inserted upstream of the mCherry coding sequence in the recipient pGMCS vector by In-Fusion cloning (Takara). The recipient plasmid constitutively expresses GFP from the P1 promoter.

### Generation and differentiation of HoxB8-immortalized myeloid progenitor cells

The procedures are described in the supplemental material.

### Proteomics

The procedures are described in the supplemental material.

### Phylogenetic distribution and genomic context of CysK2

Homologs of the *M. tuberculosis* H37Rv proteins CysK2, CysK1, CysM, Cbs, and Cds1 were identified in the UniProt database by BLAST using the corresponding *M. tuberculosis* protein sequences as queries. Close CysK2 homologs were defined as proteins sharing >50% amino acid identity with *M. tuberculosis* CysK2 over the full-length sequence. UniProt accession numbers of all proteins used for phylogenetic analysis are provided in Table S3.

Phylogenetic analysis was performed with Phylogeny.fr in advanced mode (57), using the JTT substitution model. Branch support was assessed with the Approximate Likelihood-Ratio Test (aLRT), using the minimum of SH-like and Chi2-based support values. Trees were visualized and annotated using iTOL (58).

The genomic environment of *cysK2* was examined in the IMG/M database, and the presence of genes predicted to be involved in copper homeostasis in the vicinity of *cysK2*, including multicopper oxidases, copper chaperones, and copper-transporting P-type ATPases, was recorded and compared across species.

### Secondary ion mass spectrometry

For ^13^C labeling, *M. tuberculosis* was grown in 7H9 medium supplemented with ADC, 0.05% Tyloxapol, and 10 g/L ^13^C_3_-glycerol (Sigma; 99 atom % ^13^C). Bone marrow-derived macrophages were seeded on indium tin oxide (ITO)-coated glass coverslips (PGO, Präzisions Glas & Optik GmbH, Germany) as previously described (35). Cells were infected with ^13^C-labeled bacteria at a multiplicity of infection (MOI) of 1. Following a 4-h incubation at 37°C in RPMI GlutaMAX HEPES medium (Gibco) supplemented with 10% FBS, the infected cells were washed three times with DPBS. The culture medium was then replaced with fresh medium containing 10% FBS and 200 μM CuCl_2_. Three days post-infection, cells were fixed with 4% paraformaldehyde for 2 h and subsequently washed three times with distilled water.

After drying in air at room temperature, the ITO glass slices were introduced into a NanoSIMS-50 Ion microprobe (CAMECA, Gennevilliers, France) operated in scanning mode (59, 60). The detailed analytical conditions for SIMS imaging were described previously (61). For this study, a tightly focused Cs^+^ primary ion beam steps over the surface of the sample and five secondary ion species (^12^C^−^, ^13^C^−^, ^12^C^14^N^−^, ^31^P^−^, and ^63^Cu^−^) were monitored in parallel to create images of these selected ion species. More specifically, the detection of ^63^Cu^−^, with a precise mass of 62.92959 amu, requires moderate mass resolution conditions so as to prevent mass interference by neighboring ion species (not only the very intense ^31^P^16^O_2_, 62.96358 amu, at a relative mass distance, M/ΔM, of 1,852, but also an abundant ^31^P^32^S, 62.94583 amu, much closer, at a M/ΔM of 3,875).

During the analysis, the primary beam intensity was 1 pA with a typical probe size of 100 nm and the raster size (field of view) was about 30 μm with an image definition of 512 × 512 pixels. The image acquisition was carried out using multiframe mode with a dwell time of 0.5 ms per pixel per frame, and up to 100 frames were acquired so as to allow the accumulation of the weak ^63^Cu^−^ signal. The overall counting time was then 50 ms per pixel and the total analysis time was about 3 h 40 min.

Image processing was performed using ImageJ software (62) by means of OpenMIMS plug-in (63). After raw data extraction, multiframe images were properly aligned using ^12^C^14^N^−^ images as reference before a summed image was obtained for each ion species. A map of ^13^C atomic fraction was then derived from ^12^C^−^ and ^13^C^−^ images as ^13^C/(^12^C + ^13^C). The location of bacteria inside the host cell was highlighted by high ^13^C composition. The distribution of ^31^P^−^ and ^63^Cu^−^ was shown based on the respective number of ion counts per pixel. These elemental and isotopic maps were displayed using Fire mode LUT (lookup table) in ImageJ. Further, color overlay images were generated using ImageJ to highlight the location of ^63^Cu^−^ spots inside the bacteria, as well as the colocalization of ^63^Cu^−^ and ^31^P^−^ spots.

### RNA extraction and RT-qPCR

For RT-qPCR, total RNA was isolated and cDNA synthesized as previously described (35).

Quantitative real-time PCR was performed using gene-specific primers (Table S2) and TB Green Premix Ex Taq (Takara) on an ABI Prism 7500 instrument (Applied Biosystems). Data were analyzed with 7500 software v2.0.6 (Applied Biosystems) and expressed as ΔCt values relative to the reference gene *rpoB* for bacterial samples or *Actb* for eukaryotic samples.

### RNA sequencing

The *M. tuberculosis* H37Rv strain was grown in triplicate in modified copper-free 7H9 medium supplemented with either no added copper, 20 µM CuSO_4_, or 100 µM CuSO_4_. Total bacterial RNA was extracted from 10 mL cultures harvested at logarithmic phase (OD_600_ = 0.8).

Bulk RNA sequencing was performed by Single Cell Discoveries (Utrecht, The Netherlands) using an adapted version of the VASA-seq protocol as previously described (35).

### Macrophage infection

Bone marrow cells were isolated from the femurs and tibias of C57BL/6 mice and differentiated into macrophages as previously described (35). Cells were infected with *M. tuberculosis* at a MOI of 1 for 4 h at 37°C. Following infection, cells were washed three times with DPBS and incubated in fresh medium supplemented with the indicated concentrations of CuSO_4_. At the specified time points post-infection, cells were washed twice with DPBS and fixed for 2 h at room temperature in either 4% paraformaldehyde (PFA) in DPBS or, for strains expressing the Mrx1-roGFP reporter, in DPBS containing 4% PFA and 10 mM N-ethylmaleimide (NEM). Fixed samples were washed three times with DPBS before analysis by confocal laser scanning microscopy (Zeiss LSM710).

For strains expressing Mrx1-roGFP, the intracellular redox state was determined by calculating the ratio of the oxidized to reduced forms of the probe on a pixel-by-pixel basis. This ratio was obtained by dividing the fluorescence intensity following excitation at 405 nm by that following excitation at 488 nm, with emission collected between 510 and 540 nm. Image analysis was performed using ImageJ software.

### Mouse infection and CFU enumeration

Female C3HeB/FeJ or C57BL/6 mice, 6–8 weeks of age, were infected by aerosol using a DSI Buxco Inhalation Tower. Mice were exposed for 17 min to aerosols generated from a 5 mL bacterial suspension containing 2 × 10^6^ bacilli. At 7, 21, and 42 (C57BL/6) or 60 (C3HeB/FeJ) days post-infection, five mice per strain were euthanized and lungs were collected. Lung homogenates were plated on Middlebrook 7H11 agar supplemented with OADC, and bacterial burden was determined by CFU enumeration.

### Histology

C3HeB/FeJ mice were infected by aerosol with approximately 100 CFU of H37Rv, Δ*cysK2*, or the complemented strain Δ*cysK2::lpqS-cysK2*, each expressing the Mrx1-roGFP redox reporter. At 60 days post-infection, mice were euthanized by intraperitoneal injection of 50 µL Euthasol (400 mg/mL sodium pentobarbital). Lungs were perfused intratracheally with 1 mL of 2% agarose (UltraPure LMP Agarose; Invitrogen) and fixed for 24 h at 4°C in periodate-lysine-paraformaldehyde. Fixed lungs were cryoprotected by sequential incubation in 10%, 20%, and 30% sucrose prepared in 0.1 M phosphate buffer, embedded in optimal cutting temperature compound (OCT), and frozen. Sections of 20 µm thickness were cut on a cryostat and analyzed by confocal laser scanning microscopy (Zeiss LSM710).

### Disk diffusion assay

*M. tuberculosis* cultures were grown to exponential phase and diluted to an OD_600_ of 0.1 in 3 mL of prewarmed top agar (7H9 containing 6 mg/mL agar). The bacterial suspension was overlaid onto Middlebrook 7H10 agar plates. After solidification, a sterile filter paper disk was placed on the surface of the top agar and loaded with 5 µL of 1 M CuSO_4_. Plates were incubated under normoxic (21% O_2_) or hypoxic (10% O_2_) conditions, and inhibition zones were measured. Values were normalized to the inhibition zone obtained for the wild-type strain under normoxia.

### Statistical analyses

Statistical analyses were performed using GraphPad Prism v10.2.3 (GraphPad Software). The statistical tests used for each experiment are indicated in the corresponding figure legends.

## Data Availability

The RNA-seq data set is accessible through the ArrayExpress collection in BioStudies with the identifier E-MTAB-17251. The mass spectrometry proteomics data have been deposited to the ProteomeXchange Consortium via the PRIDE partner repository with the data set identifier PXD076718.

## AFTER EPUB

*[This article was published on 8 September 2026 with an error in the surname of Gérald Larrouy-Maumus. The name was corrected in the current version, posted on 21 September 2026.]*

## SUPPLEMENTAL MATERIAL

Supplemental material mbio.00816-26-s0001.docx Fig. S1-S7; Tables S1-S3; supplemental methods.

ASM does not own the copyrights to Supplemental Material that may be linked to, or accessed through, an article. The authors have granted ASM a non-exclusive, world-wide license to publish the Supplemental Material files. Please contact the corresponding author directly for reuse.
