# Supplementary material for: CysK2 couples copper sensing to redox adaptation in Mycobacterium tuberculosis
Source: mBio. 2026 Sep 8;17(10):e00816-26. doi: 10.1128/mbio.00816-26 (PMC13712839; doi:10.1128/mbio.00816-26)
Supplement: Supplementary file 1 [file mbio.00816-26-s0001.docx]

**Figure S1. Copper ions induce the expression of the *cysK2* locus but not of other genes related to sulfur metabolism.** *(A)* The P1-GFP *lpqSp*-mCherry reporter strain was incubated in PBS in the presence of 0, 2, 6, 20, or 200 µM CuSO₄ for 24 h or 48 h. The mCherry/GFP fluorescence ratio was then measured by flow cytometry *(B)* *M. tuberculosis* bacteria were incubated in M9 minimal medium in presence of 0, 2, 6, 10, 20, 100 or 200 µM CuSO_4_. After 24 or 48 hours, total RNAs were extracted and genes expression was quantified by RT-qPCR. Data represent the mean ± SD of fold change in genes expression compared to the reference gene *rpoB* and the condition 0 µM CuSO_4_ from two independent experiments.

**Figure S2. Generation of CTR1- and ATP7A-deficient mouse macrophages**. *(A)* Schematic construction and differentiation of CRISPR-edited HoxB8 cell lines. Created in [https://BioRender.com](https://biorender.com/). *(B) (C)* Quantification of CTR1 and ATP7A proteins abundance in WT (Luc) and corresponding CRISPR-edited HoxB8-differentiated macrophages by proteomics. Data represent raw intensity *(B)* and percentage expression relative to WT expression *(C)*. *(D)* Western blot analysis of ATP7A in WT and ATP7A-KO clone a (used in this study) and clone b HoxB8 progenitors or HoxB8-differentiated macrophages.

**Figure S3. Hypoxia does not boost copper-mediated *cysK2* induction in *M. tuberculosis in vitro.*** *M. tuberculosis* P1-GFP *lpqSp*-mCherry reporter strain was incubated in PBS in presence of 0, 2, 6, 20 or 200 µM CuSO_4_. At different time post-incubation, bacteria were fixed and mCherry/GFP ratio was quantified by flow cytometry and normalized to the condition 0 µM CuSO_4_. Data come from two independent experiments (experiment 1: days 1 and 2; experiment 2: days 3 and 6).

**Fig. S4.** **Construction and validation of *M. tuberculosis* ∆*cysK2* deleted strain and complemented strain**. *(A)* Schematic representation of ∆*cysK2* mutant construction strategy. The allelic exchange substrate (AES) consisting in a zeocin resistance cassette flanked by two identical dif sites and the up- and downstream fragments of the target *cysK2* gene recombines with H37Rv genome and replaces the *cysK2* gene resulting in a ∆*cysK2::dif4-zeo-dif4* mutant (1). The two dif sites recombine, leading to the loss of the zeocin cassette and to the ∆*cysK2::dif4*=∆*cysK2* mutant (2). *(B)* Confirmation of *cysK2* deletion. PCR was done with primers amplifying a 1.7 kb fragment in the wild-type strain (lane 1) and a 0.7 kb fragment in the ∆*cysK2* mutant (lane 2). M: molecular size marker. *(C)* RT-qPCR quantification (∆Ct values *vs. rpoB* reference gene) of *mmcO*, *lpqS* and *cysK2* expression in *M. tuberculosis* H37Rv, ∆*cysK2* mutant and ∆*cysK2::lpqS-cysK2* complemented strain. No *cysK2* complementary DNA was amplified from ∆*cysK2* extract. Data represent mean of three technical replicates.

**Figure S5. CysK2 contributes to *M. tuberculosis* survival in C57BL/6**. Mice were infected via aerosol with H37Rv, ∆*cysK2* or complemented strains (100 CFUs per mice). Lungs were collected at 7, 21, and 42 days post-infection, and bacterial burden was quantified by CFU counting. Data represent mean ± SD for n=5 mice per strain. Statistical analysis: two-way ANOVA with Tukey’s post-hoc test on log-transformed data.

**Figure S6. The Δ*cysK2* mutant is not hypersensitive to copper in liquid medium as compared to the wild-type strain.** Both strains were cultured in 7H9 liquid medium in the presence of copper. *(A)* Bacterial growth was measured over a 17-day period (McFarland). *(B)* Survival of bacteria (CFUs count on 7H11 medium) in the presence of copper over a 9-day period.

**Figure S7. CysK2 contributes to redox homeostasis both in the intracellular environment and in necrotic cores**. C3HeB/FeJ mice were infected with H37Rv, Δ*cysK2*, and complemented strains expressing the Mrx1-roGFP redox reporter. At 60 days postinfection, lungs were collected and analyzed by confocal microscopy. The 405/488 fluorescence ratio was quantified in individual bacteria located near a nucleus (intracellular) or part of a necrotic area. Violin plots show median and interquartile range. Statistical analysis: Kruskal-Wallis test followed by Dunn’s multiple-comparison test.

**Table S1: Distribution of CysK2 orthologues among orders of Actinomycetes.**

| **Order (Class Actinomycetes)** | **Number of CysK2 homologues in UNIPROT** |
| --- | --- |
| Acidothermales | ND |
| Actinomycetales | ND |
| Bifidobacteriales | ND |
| Catenulisporales | 1 |
| Cryptosporangiales | ND |
| Frankiales | 3 |
| Geodermatophilales | 0 |
| Glycomycetales | 4 |
| Jatrophihabitantales | ND |
| Jiangellales | 1 |
| Kineosporiales | ND |
| Kitasatosporales | >100 |
| Micrococcales | 2 |
| Micromonosporales | 32 |
| Motilibacterales | ND |
| Mycobacteriales | >100 |
| Nakamurellales | ND |
| Propionibacteriales | 4 |
| Pseudonocardiales | >100 |
| Sporichthyales | ND |
| Streptosporangiales | >100 |

(*ND, not detected)

**Table S2: Primers used in this study**

| **Oligonucleotide** | **Sequence 5’->3’** | **Purpose** |
| --- | --- | --- |
|  |  |  |
| *Rv0848_up-Fw* | *gcgggatatcactagtgcgcgtgctgacgatcggcc* | Δ*cysK2* mutant |
| *Rv0848_up-Rv* | *cagtcgatccacgtggagcaaggtgcggtgcgcccg* |  |
| *Rv0848_down-Fw* | *ccactgagcgtcagacccacgtgctcaatgggagcgcgcgctat* |  |
| *Rv0848_down-Rv* | *gcgttgggagctctcccagcatttcgaaaggaaaca* |  |
| *lpqS_up-Fw_IF* | *gccacgtggaaagcgggcacggggtcagcgccaggcggtg* | Δ*cysK2* cpl strain |
| *lpqS_up-Rv_IF* | *caccgcctggcgctgaccccgtgcccgctttccacgtggc* |  |
| *attB2-SD-rvcysK2* | *ggggacagctttcttgtacaaagtggaggaagacaggctgcccatgaggtcgcggcagacccg* |  |
| *attB3-rvcysK2_Rv* | *ggggacaactttgtataataaagttgttacgacaccacctgggttg* |  |
| *prom-rv0847-rv0850Fw* | *ttgtacaaaaaagcaggcttcggcgtcgtagtggcctgg* | *pGMCS-P1-GFP-P_rv0847-rv0850_-mCherry vector* |
| *IF-prom-rv0847-rv0850Rv* | *ctttgtacaagaaagctgggtcacgtgcccgctttccacg* |  |
|  | **RT-qPCR primers** |  |
| *rpoB-Fw* | *TCGTTCTCTGACCCTCGTTTC* |  |
| *rpoB-Rv* | *ACGTGCCCTTCTCGGTCATCA* |  |
| *cysK2-Fw* | *CGAGTATTTATCCGCGCAAT* |  |
| *cysK2-Rv* | *GTGTCGAAGTAGCGTTGTGG* |  |
| *Atp7a-Fw* | *AATTGCCATAGGCACAGGCAC* |  |
| *Atp7a-Rv* | *ATGGGCAGGAAAACTCCAGCA* |  |
| *Ctr1-Fw* | *ACATTACCATGCCACCTCACCA* |  |
| *Ctr1-Rv* | *CGAAGGCTCCAGCCATTTCTCCA* |  |
| *ActinB-Fw* | *CTAAGGCCAACCGTGAAAAG* |  |
| *ActinB-Rv* | *ACCAGAGGCATACAGGGACA* |  |

**Table S3: Accession numbers of proteins used for the phylogenetic analysis shown in Fig. 3A.**

| **Species** | **UniProt Accession Number** |
| --- | --- |
| *M. smegmatis* ATCC 700084 | A0R1X2_MYCS2 (CysM); A0R5I5_MYCS2 (Cds1); A0R2X8_MYCS2 (Cbs) |
| *M. abscessus* ATCC 19977 | B1MGL6_MYCA9 (CysK2); B1MFV5_MYCA9 (Cds1); B1MKR9_MYCA9 (CysK2); B1MLY9_MYCA9 (Cbs); B1MKJ1_MYCA9 |
| *M. marinum* ATCC BAA-535 | B2HL85_MYCMM (CysK1); B2HF70_MYCMM (CysM); B2HG60_MYCMM (CysK2); B2HK82_MYCMM (CysK1); B2HT76_MYCMM (Cbs) |
| *M. kansasii* ATCC 12478 | A0A7G1IC04_MYCKA; A0A1V3X6J4_MYCKA (CysK2); A0A7G1I8R6_MYCKA; A0A1V3XHI2_MYCKA (Cbs); A0A7G1IAD3_MYCKA |
| *M. fortuitum* ATCC 6841 | A0A0N9XJC1_MYCFO; A0A0N9XGY7_MYCFO (CysM); A0A0N9YME9_MYCFO (Cds1) |
| *M. vaccae* ATCC 25954 | K0VGZ0_MYCVA (Cds1); K0UXW7_MYCVA; K0UZ64_MYCVA (Cbs) |
| *M. gordonae* ATCC 14470 | A0A1X1VMA1_MYCGO (Cds1); A0A1A6B6N8_MYCGO; A0A1A6B7I0_MYCGO (Cbs) |
| *M. avium* ATCC BAA-968 | Q73Y35_MYCPA (CysK1); Q744F1_MYCPA (Cds1); Q741R3_MYCPA (CysM) |
| *M. terrae* ATCC 15755 | A0AAD1HYC3_9MYCO; A0AAD1HXV6_9MYCO (Cbs) |
| *M. aurum* ATCC 23366 | A0A3S4RRU7_MYCAU (CysM); A0A448IYF0_MYCAU (Cbs) |

**Supplementary material**

**Generation and differentiation of HoxB8-immortalized myeloid progenitor cells**

Immortalized HoxB8 progenitor cells derived from Cas9-expressing mice were maintained in myeloid medium consisting of RPMI GlutaMAX supplemented with 10% fetal bovine serum (FBS; Merck), antibiotic-antimycotic (Gibco), 20 ng/mL GM-CSF (Miltenyi Biotec), and 0.5 µM β-estradiol. Cells were passaged every 3 days.

For macrophage differentiation, HoxB8 progenitors were washed twice with PBS and seeded at a density of 5×10^5^ cells per well in 6-well plates containing complete medium (RPMI supplemented with FBS and antibiotic-antimycotic) supplemented with 20 ng/mL recombinant mouse M-CSF (Miltenyi Biotec). Fresh medium was added on days 3 and 6, and differentiated HoxB8-derived macrophages were harvested on day 7 by incubation in PBS containing 5 mM EDTA for 5 min at 37°C.

For gene targeting, individual sgRNAs were cloned into the pLenti-sgRNA vector (Addgene #71409), with an sgRNA targeting luciferase used as a negative control. Lentiviral particles carrying the corresponding sgRNAs were produced by transfecting packaging cells with the pLenti-sgRNA constructs using Lipofectamine 3000 (Thermo Fisher) and Opti-MEM (Gibco), according to the manufacturer's instructions. HoxB8 progenitor cells 2×10^5^ were transduced with lentiviral particles in 1 mL of myeloid medium supplemented with LentiBlast Premium (OZ Biosciences). After 24 h, cells were pelleted by centrifugation at 300 g for 5 min, resuspended in fresh myeloid medium containing puromycin (7 µg/mL; InvivoGen), and selected for 72 h before further use.

**Proteomics**

HoxB8-derived macrophages were cultured as described above in 24 well plates. Cells were lysed in a solution of 5% sodium dodecyl sulfate (SDS) (Invitrogen) and 50 mM triethylammonium bicarbonate, pH 8.5. Protein disulfide bridges and cysteine residues were reduced and alkylated by incubation with 10 mM Tris (2-carboxyethyl) phosphine hydrochloride (NeoBiotech) and 40 mM 2-chloroacetamide (Aldrich), 5 min at pH 8.5 and 95°C. Proteins were digested on S-trap Micro devices (Protifi) using the manufacturer’s protocol. Briefly, samples were acidified by addition of phosphoric acid (2.5% final concentration), and proteins were precipitated by addition of 6 volumes of 90% methanol in 100 mM TEAB, pH 7.5. Samples were applied on the S-Trap Micro devices and precipitated proteins were washed 6 times with 90% methanol, 100 mM TEAB, pH 7.5. Proteins were then digested by incubation with trypsin (Promega) at 1% (trypsin/protein) and 37°C overnight. The resulting peptides were successively eluted with 40 µl of 50 mM ammonium bicarbonate, 40 µl of 0.2% formic acid and 40 µl of 50% acetonitrile, 0.2% formic acid. and speed-vacuum dried. After resuspension in 0.1% formic acid, they were desalted on Affinisep C18 T1 Tips with 0.1% formic acid, eluted with 40% acetonitrile, 0.1% formic acid and then with 70% acetonitrile, 0.1% formic acid, and finally dried again. Peptides were analyzed by nanoscale liquid chromatography using an UltiMate 3000 system (Thermo Scientific) coupled to an Orbitrap Exploris 480 mass spectrometer (Thermo Scientific). One µl of each sample was injected on the analytical C18 column (75 μm inner diameter × 50 cm, PepmapC18, 2 µm, ThermofisherElectronSAS) equilibrated in 95% solvent A (5% acetonitrile, 0.2% formic acid) and 5% solvent B (80% acetonitrile, 0.2% formic acid). Peptides were eluted using a 2.5%-40% gradient of solvent B over 62 min at a flow rate of 300 nl/min. The mass spectrometer was operated in data-independent acquisition (DIA) mode with the Xcalibur software. Full scan MS1 scans were acquired on the m/z mass range 400–1008, with a resolution of 60000, an AGC target of 300% and a maximum injection time of 100 ms. For acquisition of MS2 spectra, the DIA isolation scheme consisted of 2 x 75 staggered windows (8 m/z width, 4 m/z offset) covering the 400–1000 m/z range. The resolution of MS2 was set to 15000, with an AGC target value of 1000%. The injection time was adjusted in automatic mode and the normalized collision energy was set at 30%. Raw mass spectrometry files were processed with the DIA-NN software (version DIA-NN 1.9.2) for database search in library-free search mode. Data were searched against merged mouse entries of the UniProtKB protein database (Mouse reference proteome, 55,307 uniprot entries, downloaded on 2023-04-11). The carbamidomethylation of cysteines was set as a fixed modification, whereas oxidation of methionine and protein N-terminal acetylation were set as variable modifications. Specificity of trypsin digestion was set for cleavage after K or R, and one missed trypsin cleavage sites were allowed. Peptide length range was set to 7-30, precursor m/z range to 400-1000 and fragment ion range to 100-1700. Validation of the identifications was performed by setting the precursor false discovery rate (FDR) at 1%. Heuristic protein grouping was used, and protein groups were also validated at 1% FDR. Global normalization was enabled. The report.pg_matrix.tsv report was used to extract protein abundance metrics.
